# Role of Electroencephalography in the Assessment of Cortical Responses Elicited by Music Therapy in Burn Patients Undergoing Intensive Care

**DOI:** 10.3390/s26082358

**Published:** 2026-04-11

**Authors:** Erica Iammarino, Alessia Baldoncini, Arianna Gagliardi, Laura Burattini, Ilaria Marcantoni

**Affiliations:** Department of Information Engineering, Engineering Faculty, Università Politecnica delle Marche, 60131 Ancona, Italy; e.iammarino@pm.univpm.it (E.I.); s1109927@studenti.univpm.it (A.B.); s1109228@studenti.univpm.it (A.G.); l.burattini@univpm.it (L.B.)

**Keywords:** electroencephalography, music, brain signal processing, electrophysiology

## Abstract

Music therapy (MT) is increasingly being integrated into intensive care unit (ICU) settings to modulate pain, stress, and emotional dysregulation. Although clinically promising, objective biomarkers for quantifying its neurophysiological effects are still missing. In this context, the electroencephalogram (EEG) represents a valid tool to assess cortical dynamics associated with cognitive–affective engagement elicited by MT. Our study aims to evaluate the role of electroencephalography as an objective tool for monitoring cortical responses to MT in the ICU. EEGs acquired from nine burn patients undergoing MT in the ICU were considered. Signals were preprocessed to improve the signal-to-noise ratio. Then, six frequency bands (delta, theta, alpha, beta, gamma, and sensorimotor rhythm) were extracted to compute band powers and derive 37 involvement indexes, which were statistically compared across three experimental phases: before, during, and after MT. Results demonstrate that involvement indexes effectively capture neurophysiological shifts induced by MT. Significant differences were observed in 22 indexes when comparing During-MT and Post-MT phases, with 2 indexes being statistically different also when comparing During-MT and Pre-MT phases; 5 indexes differed statistically when comparing Pre-MT and Post-MT phases. These results suggest a transient cortical engagement elicited during MT in ICU settings. Our findings align with previous research reporting EEG (and certain EEG-derived involvement indexes) sensitivity to capture music-induced cognitive and emotional modulation. This confirms electroencephalography potential to objectively reflect MT effects and support its integration in multidisciplinary burn care; however, analysis on larger cohorts is necessary to validate EEG as a clinical tool in MT.

## 1. Introduction

Music is widely recognized for its capacity to evoke a broad spectrum of emotional responses and effectively impact listeners’ moods. Thanks to these properties, it forms the foundation of music therapy (MT) interventions and it is often used as an adjunctive therapy in various clinical settings [1]. According to the American Music Therapy Association, MT is an established health profession defined as the clinical and evidence-based use of music interventions within a therapeutic relationship by a qualified professional to address individuals’ physical, emotional, cognitive, and social needs [2]. Indeed, these interventions support a variety of healthcare goals, including promoting wellness, managing stress, alleviating pain, and improving communication [2,3]. In recent years, MT is increasingly being integrated into high-stress clinical environments such as the intensive care unit (ICU), where it serves as a complementary, non-pharmacological treatment. Within the ICU, patients frequently encounter physiological and psychological stressors, including pain, anxiety, and sleep deprivation. An umbrella review of music interventions in ICU patients found that 62.5% of the non-pharmacological intervention trials to treat uncomfortable symptoms included music-based interventions [4]. A core MT technique in these clinical settings is music-assisted relaxation (MAR), which is based on the principle of entrainment and involves listening to live, improvised music combined with guided relaxation and mental imagery [5]. By matching musical elements to the behavioral and emotional states of the patient, MAR aims to induce a deep relaxation response and has been shown to be effective in improving insomnia in adults, promoting motor skills and communication in patients with brain damage, relieving depressive symptoms, and reducing anxiety and pain in various populations, such as presurgical patients, coronary patients, and cancer patients [6,7,8].

Based on the extensive evidence reviewed, the World Health Organization also recognizes the potential of arts-based interventions, including MT, to support health and well-being and highlights the importance of promoting the collaboration between the health and arts sectors [9]. Despite the observed clinical benefits, MT remains underutilized in routine clinical practice, probably because there is still a lack of objective and comprehensive validation of its effects, which are still mostly assessed through self-reported outcomes or behavioral observations. Although these measures provide valuable insights into patients’ perceived well-being, there is a growing need for objective neurophysiological measures that can offer a crucial complementary perspective for evaluating patients’ responses to MT interventions. In this context, neurophysiological measures such as electroencephalography (EEG) can help establish a scientific basis for the effect of MT in clinical settings by providing objective indicators of brain responses to musical interventions. Indeed, EEG represents a valid non-invasive tool for investigating cortical dynamics associated with cognitive and emotional engagement elicited by MT. By analyzing oscillatory activity across different frequency bands (e.g., delta, theta, alpha, beta, and gamma), EEG allows for the investigation of neural correlates of emotional processing, stress regulation, and music-induced mental engagement [10].

Previous studies examining MAR have primarily focused on changes in the spectral power of single EEG frequency bands. Specific neural patterns identified include an increased occipital alpha activity, which is associated with inward-focused states and guided imagery, and decreased frontal delta and theta activity, which may reflect a shift in attention away from pain stimuli [1,6,7,11,12]. However, relying on the spectral power of a single frequency band may be insufficient for a consistent assessment of a patient’s mental engagement. Wang et al. [13] suggested that a more robust representation of cognitive engagement can be achieved through the integration of multiple brain rhythms, such as by calculating ratios between different spectral powers. Marcantoni et al. [14] conducted a systematic review that identified 37 distinct EEG-derived ratio indexes currently used in the scientific literature to quantify human mental involvement. These indexes, which capture the interaction between high- and low-frequency oscillations, may offer a promising objective framework for evaluating the neurophysiological responses elicited by MT. Despite these advancements, EEG-based monitoring studies in MT and pain management remain largely limited to the analysis of single frequency bands and are often conducted in controlled experimental settings or non-clinical populations. Indeed, to the best of our knowledge, the use of multiple EEG involvement indexes in assessing neurophysiological responses during MT has not yet been standardized or widely validated, and their role remains unexplored within ICU settings.

The present study aims to address this gap by evaluating the potential role of electroencephalography in the assessment of cortical responses elicited by MT in adult burn patients hospitalized in the ICU. More precisely, the patients’ emotional states will be assessed before, during and after an MAR session through the analysis of brain rhythms and ratio involvement indexes.

## 2. Materials and Methods

### 2.1. Study Population

The dataset used in this study is freely available on Openneuro (doi: 10.18112/openneuro.ds004840.v1.0.1). It is part of a randomized controlled trial involving 82 adult burn patients admitted to the ICU of the University Hospital Fundación Santa Fe de Bogotá (FSFB) in Colombia. Participants were randomly allocated to either an intervention group or a control group in a 1:1 ratio. Over a two-week period, patients in the intervention group received standard medical care supplemented with up to six MT sessions, each administered by a certified music therapist [7].

Electrophysiological data were collected from a subset of 9 patients in the intervention group, corresponding to approximately 11% of the total enrolled population. Participants were considered eligible for electrophysiological monitoring if they were adult patients with an expected hospital stay longer than 7 days, with no pre-existing psychiatric disorders, cognitive impairments, sedation, mechanical ventilation, and burn injuries involving the head or neck. Acquisitions were performed during an MT intervention, consisting of:

- Pre-MT phase: the patient remained at rest, either with eyes closed or visually fixed on a point (mean duration of 533 ± 134 s);
- During-MT phase: the MT intervention was actively administered by a qualified therapist (mean duration of 1202 ± 331 s);
- Post-MT phase: the patient recovers after the MT intervention without any specific instructions (mean duration of 576 ± 300 s).

EEG data were acquired using the Micromed LTM64 system (Micromed S.p.A., Treviso, Italy), with a sampling frequency of at least 256 Hz (variable among EEG acquisitions). Electrode placement followed the international 10–20 system, but to reduce the setup time for patients due to their medical condition, the number of electrodes was reduced to eight: FP1, FP2, T3, T4, C3, C4, O1, and O2. The reference electrode was positioned at Cz, while the ground electrode was placed on the mastoids, located behind each ear on the temporal bone.

In addition to EEG signals, for some patients, electrocardiogram (ECG) and electromyogram (EMG) signals were simultaneously recorded. In addition, to correlate electrophysiological data with subjective clinical outcomes, the following questionnaires were administered before the Pre-MT phase and after the Post-MT phase:

- Visual Analog Scale (VAS) for pain assessment. The scale ranges from 0 (no pain) to 10 (maximum pain).
- Hospital Anxiety and Depression Scale (HADS), consisting of two subscales (A-HADS for anxiety and D-HADS for depression), each with seven items scored on a four-point Likert scale (0–3). Each subscale yields a maximum score of 21, with higher scores indicating greater symptom severity.

Details about the study population, including age, sex, and participants’ scores to VAS and HADS questionnaires, are reported in Table 1 [7].

### 2.2. Data Processing

Signal processing was performed in Matlab R2024b using EEGLAB toolbox (version 2025.0.0). EEG recordings originally acquired at sampling frequencies higher than 256 Hz were first downsampled to 256 Hz to ensure consistency across participants’ data. Then, EEG preprocessing included average re-referencing, band-pass filtering between 0.5 and 50 Hz using a finite impulse response (FIR) filter, and artifact removal by independent component analysis (ICA). Independent components (ICs) obtained from ICA decomposition were automatically classified with the ICLabel algorithm [15]. This classifier assigns each component to one of seven classes: four physiological sources (“brain”, “muscle”, “eye”, and “heart”), two non-physiological sources (“line noise” and “channel noise”), and an “other” class for components that do not clearly match predefined patterns. Components were marked for rejection if their probability of belonging to the “brain” class was below 10%, or if the probability associated with any non-brain class exceeded 85%. ICs marked as artifact-related were subsequently removed, and the retained ICs were used to reconstruct cleaner EEG signals. Following artifact correction, the continuous EEG recordings were segmented according to the experimental protocol into three distinct phases: prior to the MT intervention (Pre-MT), during the MT intervention (During-MT) and immediately after the MT intervention (Post-MT). All subsequent analyses were performed separately within each protocol phase.

EEG signals were divided into overlapping epochs. Specifically, 20 s epochs were extracted in a sliding-window manner with a 2 s step size, until the end of the recording was reached. Within each epoch, a channel-quality check was performed. The channel-quality check consisted of dividing each epoch signal into consecutive 1 s segments. Within these 1 s segments, a value of four times the standard deviation of the segment amplitude (for each channel) was computed. If this value exceeded 100 µV in more than three segments within the same epoch, the corresponding EEG channel was rejected from that epoch.

Spectral analysis was then performed on each EEG epoch. Band-limited signals were obtained using a 6th-order bidirectional Butterworth filter to isolate the following frequency bands: delta (1–4 Hz), theta (4–8 Hz), alpha (8–12 Hz), sensorimotor rhythm (SMR, 12–15 Hz), beta (15–30 Hz), and gamma (>30 Hz). For each band, the power spectral density (PSD) was estimated using Welch’s overlapped segment averaging method with a Hamming window of 4 s and a 50% overlap. The band power was then quantified as the area under the PSD curve computed with the trapezoidal rule. Based on these spectral energy estimates, 37 involvement indexes were calculated. Each index was defined as a ratio between the spectral energy of two or more frequency bands. The explicit mathematical formulations of these ratio indexes are reported in Table 2 [14].

### 2.3. Statistics

EEG-derived features (i.e., band powers and involvement indexes) were first averaged by computing the median value across all epochs belonging to the same protocol phase, resulting in a single representative value per patient and per phase. Then, to assess their topographical distribution, channels were grouped into four brain regions (i.e., frontal, temporal, central, and occipital), and feature values were averaged (mean) across channels within the same brain region. The resulting distributions were visually represented across the three protocol phases using box plots, which make it possible to evaluate median shifts and interquartile ranges throughout the MT session.

Statistical comparisons were performed for each feature and brain region. First, the non-parametric Friedman test was employed to evaluate global differences in EEG features across the three protocol phases (Pre-MT, During-MT, and Post-MT). Then, pairwise comparisons between all combinations of the protocol phases were conducted using the Wilcoxon signed-rank test. In both tests, the Benjamini–Hochberg false discovery rate (FDR) procedure was applied to adjust for multiple comparisons, and statistical significance *p* was set to 0.05 after correction.

Changes in subjective pain and psychological distress were evaluated by comparing pre- and post-intervention scores from the VAS and the HADS using the Wilcoxon signed-rank test, setting the statistical significance *p* at 0.05. Subsequently, the relationship between these subjective measures and brain powers was explored using Spearman’s rank correlation (*ρ*). To determine if changes in EEG-derived measures were associated with reductions in self-reported anxiety or pain, the correlation analysis focused on the change in both measures between the Post-MT phase and the Pre-MT phase, computed as the arithmetic difference between post-intervention and pre-intervention values.

## 3. Results

**Figure 1:**
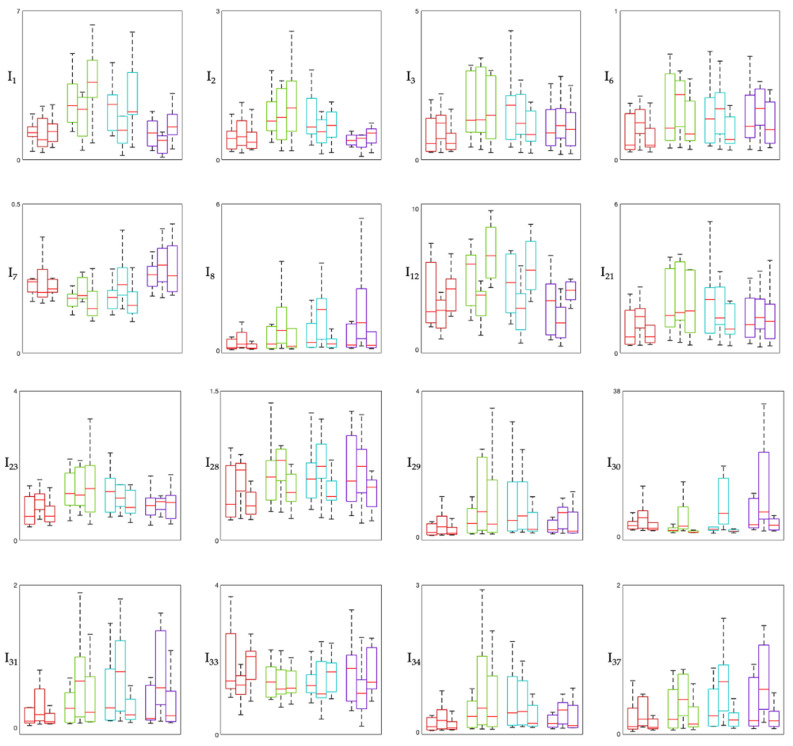
Box plot showing high-frequency-based involvement indexes. The figure displays indexes having high-frequency rhythms in the numerator in their definition (see Table 1). In each panel, boxes are color-coded by brain region: red boxes refer to frontal region, green boxes to temporal region, cyan boxes to central region, violet boxes to occipital region. Within each region, the first box refers to the Pre-MT phase, the second to the During-MT phase, and the third to the Post-MT phase. The central horizontal line in each block is the median, while the box borders represent the interquartile range.

**Figure 2:**
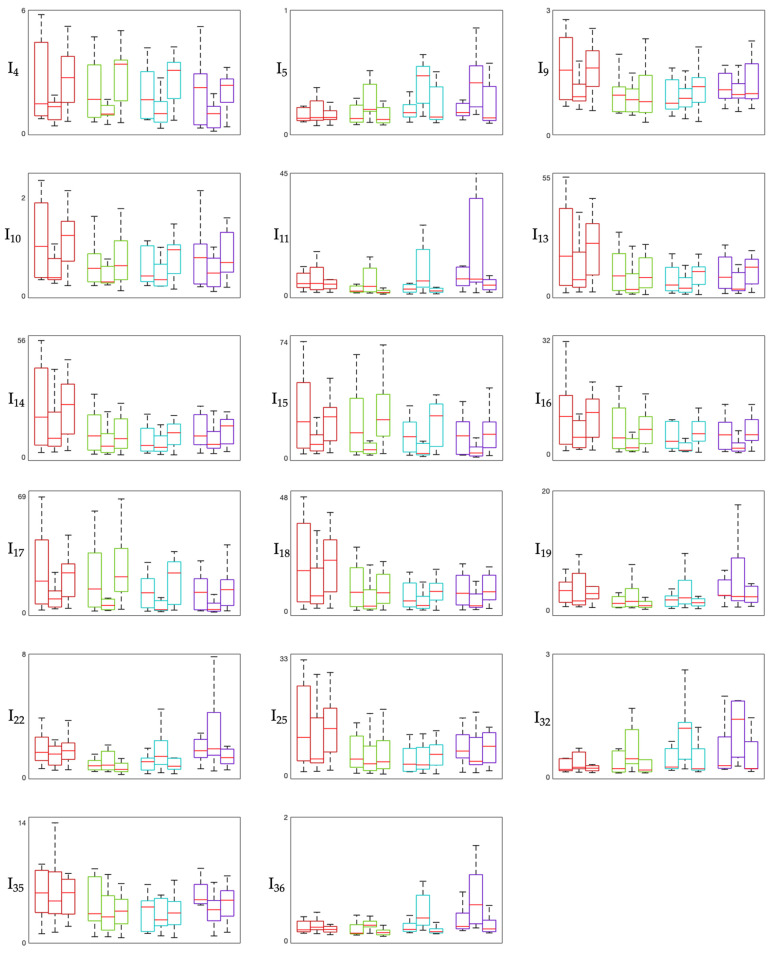
Box plot showing slow-wave-based involvement indexes. The figure displays indexes having slow-wave rhythms in the numerator in their definition (see Table 1). In each panel, boxes are color-coded by brain region: red boxes refer to frontal region, green boxes to temporal region, cyan boxes to central region, violet boxes to occipital region. Within each region, the first box refers to the Pre-MT phase, the second to the During-MT phase, and the third to the Post-MT phase. The central horizontal line in each block is the median, while the box borders represent the interquartile range.

**Figure 3:**
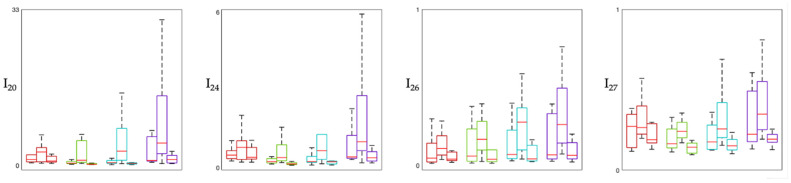
Box plot showing alpha-based involvement indexes. The figure displays indexes having alpha rhythm in the numerator in their definition (see Table 1). In each panel, boxes are color-coded by brain region: red boxes refer to frontal region, green boxes to temporal region, cyan boxes to central region, violet boxes to occipital region. Within each region, the first box refers to the Pre-MT phase, the second to the During-MT phase, and the third to the Post-MT phase. The central horizontal line in each block is the median, while the box borders represent the interquartile range.

Figure 1, Figure 2 and Figure 3 show the distribution of involvement indexes across the three experimental phases (Pre-MT, During-MT, and Post-MT) for each brain region. Specifically, involvement indexes having high-frequency rhythms (β and γ, also when summed to other rhythms) in the numerator are shown in Figure 1, involvement indexes having slow-wave rhythms (δ, and θ, also when summed to α) in the numerator are shown in Figure 2, and involvement indexes having α rhythm in the numerator are shown in Figure 3. In each figure, each panel corresponds to one involvement index, with colors identifying the different brain regions: the frontal region is displayed in red, the temporal region in green, the central region in cyan, and the occipital region in violet. Each box plot displays the median (horizontal red line) and the interquartile range (box borders) of the index distribution over the population.

The results of the statistical analysis are summarized in Table 3. The “Global variance” column reports the results of the Friedman test, indicating features that showed significant changes across the entire protocol. Subsequent columns detail the pairwise comparisons between phases (Pre-MT vs. During-MT, During-MT vs. Post-MT, and Pre-MT vs. Post-MT), analyzed via Wilcoxon signed-rank tests. More specifically, each cell indicates the brain region where the feature reached statistical significance after FDR correction (*p* < 0.05); the designation “all” implies that the effect was consistent for all the four brain regions considered. Overall, pairwise comparisons revealed that a total of 24 involvement indexes (65%) exhibited unique significant differences across the protocol phases. More specifically, 22 indexes differed statistically when comparing During-MT and Post-MT phases, while 4 indexes differed statistically when comparing During-MT and Pre-MT phases. Of these, two indexes were statistically different in both comparisons.

When comparing VAS and HADS scores given by the patients before and after the MT intervention, no statistically significant difference was found. Spearman’s rank correlation analysis revealed some possible patterns linking subjective responses to neurophysiological changes (Table 4). Moderate-to-strong correlations are highlighted in bold and were observed between changes in VAS and alpha power in the occipital region, changes in A-HADS and both alpha and gamma powers in the central region, changes in D-HADS and alpha powers in the frontal region, and changes in D-HADS and both beta and gamma powers in the central region.

## 4. Discussion

This study investigated the role of electroencephalography in assessing the cortical responses and effects of MT in adult burn patients undergoing intensive care. To this aim, we conducted an analysis of the dataset published by Cordoba-Silva and colleagues, consisting of EEG recordings acquired from nine patients hospitalized in the ICU. The fact that the EEG data were acquired before, during and after an MAR session makes it suitable for characterizing phase-dependent cortical dynamics and for assessing the short-term efficacy of MT interventions in a critical clinical setting [7].

The objective neurophysiological assessment of ICU patients through EEG presents several technical challenges, primarily due to the high levels of environmental electrical noise and physiological artifacts. In the ICU, unlike other recording environments, there can be numerous electrical signal generators, such as bed oscillators, ventilators, temperature management devices, hemodialysis and infusion pumps. Electrical interference from this medical equipment may affect signal quality and reduce the reliability of the extracted features. To face this, a customized EEG preprocessing pipeline was implemented.

After standard filtering, artifact identification and removal was achieved using ICLabel [15], a peer-reviewed tool for EEG independent component classification which allowed for the objective and reproducible isolation of non-brain sources (such as ocular movements, cardiac interference, and muscle activity) without the bias of manual inspection and guaranteeing the reproducibility of the procedure. To capture residual artifacts not necessarily attributable to a specific pattern of clearly recognizable origin and included among ICLabel classes (which have likely persisted after ICA), an additional self-implemented algorithm for bad channel detection based on signal standard deviation was applied. The algorithm for channel-quality check was already implemented and tested in another condition very different in terms of context (that is sport application) but similar in terms of criticality for the EEG quality [16]. Specifically, by using 1 s segments, this procedure enables the detection of transient artifacts that may affect only brief portions of the signal but could still compromise data quality and reliable results. Furthermore, the implementation of a sliding-window epoching strategy (i.e., 20 s windows with a 2 s step) allowed us to capture the temporal evolution of the EEG signal, while the subsequent averaging of these epochs within protocol phases provided a stable steady-state representation of each condition.

From the distribution of involvement indexes across the three experimental phases in the different brain regions considered (Figure 1, Figure 2 and Figure 3), we can observe that most indexes exhibit variations across phases, with the most pronounced changes in median values and interquartile ranges observed between the Pre-MT and During-MT conditions in multiple regions. For what concerns involvement indexes having beta in the numerator (Figure 1), they tend to display lower median values during the MT phase compared with the baseline condition, especially in the temporal and central regions. This pattern may reflect a relative reduction in beta activity during the intervention phase, possibly suggesting a reduced cortical arousal, given that beta activity is typically associated with high arousal and active cognitive processing [17,18]. Indexes having beta over gamma in their definition (e.g., I_28_ = β/(θ + γ), I_30_ = (α + β)/γ) showed a marked increase in the During-MT phase. Since gamma activity appears in the denominator, this increase may indicate a relative suppression of high-frequency activity, often associated with active sensory processing. This trend is also confirmed by the statistical analysis, which reveals significant differences across all regions, especially when comparing the During-MT and Post-MT phases. Indexes having alpha in the numerator in their definition showed increased values during the MT with respect to both the baseline and the Post-MT phase (Figure 3). This may reflect a state of coordinated neural activity in the alpha frequency band, also referred to as alpha synchronization [19], where the brain shifts from external stressors toward a more introspective relaxed state during the intervention. Indexes having primarily delta and theta in the numerator in their definition (Figure 2), reflecting deep internal states and subconscious processing, showed a characteristic U-shaped pattern. Indeed, many of these indexes exhibited higher values during the Pre-MT and Post-MT phases and lower values during the MT phase, possibly reflecting task-related modulation of slow oscillatory activity. This is in accordance with previous studies on brain rhythm powers, which reported significant differences in both delta and theta powers during MT, possibly linked to the modulation of pain perception [7,20]. It is also consistent with prior physiological interpretations of indexes such as I_16_ ((δ + θ)/(α + β)), which has been specifically utilized to evaluate music effects using EEG signal processing [21]. Regarding cortical localization, most indexes show consistent behavior across brain regions, with the temporal and central regions being those with the greatest number of significant changes. This suggests that the auditory processing and sensory-motor integration areas are the primary drivers of the early spectral response to the MT intervention [22].

The results of the statistical analysis showed that most of the significant differences emerged when comparing the During-MT and the Post-MT phases, while no statistically significant differences were observed between the Pre-MT and Post-MT phases. The fact that nearly 90% of the indexes returned to baseline levels immediately following the MAR session may suggest that involvement indexes are able to reflect transient cortical engagement, capturing the concurrent effects associated with the ongoing therapeutic experience; rather, they are not able to reflect possible short-term changes detectable at the pre–post level (on the long-term effect, the protocol does not allow any observation). At the same time, we cannot rule out the possibility that it is the MT intervention itself that primarily has a short-term effect. Further investigations are needed to determine whether repeated sessions or longer observation periods could reveal more persistent neurophysiological effects. Once their role in reflecting the effect of MT has been possibly confirmed, they could also make an important contribution in guiding the therapeutic approach (duration of intervention, frequency of repetition, etc.).

To further contextualize our findings, it is worth considering the physiological basis of EEG-derived involvement indexes. These measures capture the dynamic interplay between the brain’s inhibitory states, typically associated with slow-wave delta, theta, and alpha oscillations, and its excitatory or “active” states, driven by beta and gamma oscillations. Historically, the most widely adopted indexes are those first introduced by Pope et al. in 1995 [23], which correspond to I_1_ (β/α), I_2_ (β/(θ + α)), and I_3_ (β/θ) reported in Table 1. While these ratios are the established “gold standard” for monitoring arousal and cognitive load in healthy populations, at the current stage of research on involvement indexes, there is no clear consensus on which involvement indexes are most clinically relevant, as their interpretation is likely to be strongly context-dependent. Further proving this, our results revealed that Pope’s indexes were not the most statistically sensitive indicators for adult burn patients undergoing MAR. This observation confirms that a broad exploratory approach involving a multidimensional evaluation of all the 37 indexes is warranted to identify those that are most suitable for assessing engagement and neurophysiological effects in MT interventions. This contributes to the identification of markers that may be more responsive to the effects of MT in this specific population.

The relationship between self-reported psychological responses and EEG-derived biomarkers was further elucidated through Spearman’s rank correlations, linking spectral power fluctuations to changes in perceived pain, anxiety, and depression. The analysis focused on the spectral power of single frequency bands, rather than composite indexes, to allow for a more direct and biologically interpretable mapping of neural mechanisms and psychological state. A moderate negative correlation was observed between VAS (pain) and alpha power across the occipital region, indicating that a reduction in perceived pain may be associated with a rise in alpha synchronization and supporting the role of alpha rhythm as a potential neurophysiological marker of pain modulation [24]. Regarding psychological distress assessed by the HADS, the strong positive correlation with central beta and gamma in the central region suggests that a reduction in the perceived anxiety and depression is reflected in a reduced high-frequency rhythm power. Interestingly, delta and theta rhythms showed weaker correlations with psychological scales, suggesting that high-frequency bands are more sensitive biomarkers for changes in emotional states experienced during MT in this population.

Some limitations of this study should be acknowledged. The primary limitation is the small sample size, which prevented robust statistical power. In relation to this aspect, we should also consider the methodological and clinical constraints of the particular setting addressed; indeed, not all ICU patients can be eligible for monitoring based on biomedical signals, as the ICU setting imposes significant restrictions, including unstable clinical conditions, sedation, mechanical ventilation, and burn injuries affecting areas possibly required for electrode placement. Moreover, the demographic composition of the sample, consisting of eight males out of nine participants and a mean age of 33 years, may limit the applicability of the findings across the broader population. Inter-patient variability represents another limitation of the study. Indeed, burn patients may also differ in terms of injury severity, pain levels, medication, and psychological state, all of which can influence EEG signals and engagement-related metrics. Hence, this study has to be intended as the observation of a small cohort of patients without the purpose of generalizing the obtained outcomes, but rather providing first insights about the potential use of involvement indexes for further research and identifying potential trends that may guide future investigations with larger samples. Future studies should perform analogous analyses of the involvement indexes on larger databases in order to possibly confirm our preliminary results and benefit from them as a valid term of comparison. In addition, when working with burn patients, practical aspects related to EEG acquisition must be taken into account. Applying a full set of electrodes can be difficult due to the presence of burns, dressings, or sensitive skin areas. Future studies should therefore explore different electrode configurations, including reduced or region-specific montages, that can ensure reliable monitoring while remaining feasible in clinical settings. Identifying flexible electrode configurations adapted to the location and extent of burns could facilitate the implementation of EEG monitoring protocols in this population.

## 5. Conclusions

Electroencephalography can support the objective assessment and monitoring of cortical responses elicited by music therapy in adult burn patients undergoing intensive care. Our results provide preliminary evidence that EEG involvement indexes are able to reflect the transient cortical engagement that occur during music therapy sessions. Thus, they suggest the potential use of electroencephalography as a complementary tool in multidisciplinary burn care. However, the small sample size and the lack of homogeneity in terms of age and gender limit the generalizability of the results, so further research is needed.

## Figures and Tables

**Table 1:** General characteristics of the study population. Total values are expressed as mean ± standard deviation for continuous variables, and as number of occurrences for categorical ones.

Patient	Age	Sex	VAS		A-HADS		D-HADS	
			Pre-MT	Post-MT	Pre-MT	Post-MT	Pre-MT	Post-MT
**1**	36	M	0	0	9	4	7	2
**2**	20	M	3	0	9	8	3	2
**3**	65	M	0	0	2	2	3	0
**4**	29	M	2	0	7	6	7	1
**5**	18	F	3	2	11	9	6	9
**6**	19	M	0	0	7	1	4	1
**7**	58	M	2	0	0	1	0	2
**8**	28	M	1	0	2	1	0	0
**9**	29	M	0	1	4	–	2	–
**Total ^1^**	33 ± 17	8 M/1 F	1 ± 1	0 ± 1	6 ± 4	4 ± 3	4 ± 3	2 ± 3

F: female, M: male, –: missing information. ^1^ Mean A-HADS and D-HADS were computed excluding patient 9.

**Table 2:** Mathematical formulation of the involvement indexes [14].

Index	Formula	Index	Formula
I_1_	β/α	I_20_	α/γ
I_2_	β/(θ + α)	I_21_	(SMR + β)/θ
I_3_	β/θ	I_22_	(θ + α)/(β + γ)
I_4_	θ/α	I_23_	(α + β)/(θ + α)
I_5_	θ/δ	I_24_	α/(β + γ)
I_6_	SMR/θ	I_25_	(δ + θ + α)/(β + γ)
I_7_	SMR/β	I_26_	α/(δ + θ + α)
I_8_	(α + β)/δ	I_27_	α/(θ + α + β)
I_9_	(θ + α)/(α + β)	I_28_	β/(θ + γ)
I_10_	θ/(α + β)	I_29_	(β + γ)/δ
I_11_	(θ + α)/γ	I_30_	(α + β)/γ
I_12_	(θ + β)/α	I_31_	(α + γ)/(δ + θ)
I_13_	(δ + θ)/β	I_32_	(θ + α)/δ
I_14_	(δ + θ + α)/β	I_33_	(θ + β)/(α + γ)
I_15_	(δ + θ)/α	I_34_	(β + γ)/(δ + θ)
I_16_	(δ + θ)/(α + β)	I_35_	(δ + α)/(θ + γ)
I_17_	δ/α	I_36_	(θ + α)/(δ + β + γ)
I_18_	δ/β	I_37_	(α + β)/(δ + θ + γ)
I_19_	θ/γ		

**Table 3:** Statistical analysis outcomes. Each cell indicates the brain region where the feature was statistically different when comparing the protocol phases.

Feature	Global Variance	Pre-MT vs. During-MT	During-MT vs. Post-MT	Pre-MT vs. Post-MT
Alpha power				
Beta power	F, T, C		all	
Gamma power	T, C, O		all	
Delta power	C	all	all	
Theta power	T, C		T, C	
I_1_	T, C		T, C, O	
I_2_		F		
I_3_				
I_4_	T, C		all	
I_5_	C, O		T, C, O	
I_6_				
I_7_		C		
I_8_				
I_9_			F	
I_10_			F, C	
I_11_	O		O	
I_12_	T, C		all	
I_13_				
I_14_				
I_15_	C	T, C	all	
I_16_			C, O	
I_17_	C	T, C	T, C, O	
I_18_			O	
I_19_				
I_20_	T, C, O		all	
I_21_				
I_22_	O		C, O	
I_23_			F	
I_24_	T, C, O		all	
I_25_				
I_26_	C		T, C, O	
I_27_	T, C		T, C, O	
I_28_	T, C		F, T, C	
I_29_				
I_30_	T, C, O		all	
I_31_				
I_32_	C, O		T, C, O	
I_33_				
I_34_				
I_35_				
I_36_	T, C, O		T, C, O	
I_37_	C		T, C	

F: frontal; T: temporal; C: central; O: occipital.

**Table 4:** Spearman’s correlations between questionnaire scores (i.e., VAS, A-HADS, D-HADS) and rhythm powers computed for each brain region.

EEG Rhythm	Frontal	Temporal	Central	Occipital
**VAS (Pain)**				
Alpha	−0.50	−0.24	−0.25	**−0.57**
Beta	0.36	0.34	0.30	−0.33
Gamma	0.44	0.42	0.15	0.22
Delta	0.50	0.50	0.50	0.40
Theta	0.35	0.19	0.34	0.35
**A-HADS (Anxiety)**				
Alpha	0.39	0.12	**0.86**	0.12
Beta	0.39	0.10	0.51	−0.17
Gamma	0.54	0.10	**0.66**	−0.05
Delta	−0.02	−0.05	−0.05	0.02
Theta	0.10	−0.07	−0.12	−0.37
**D-HADS (Depression)**				
Alpha	**0.67**	0.13	0.23	−0.54
Beta	0.54	0.34	**0.61**	−0.2
Gamma	0.44	0.34	**0.78**	−0.17
Delta	0.42	0.33	0.32	0.32
Theta	0.37	0.36	0.36	0.18

Moderate-to-strong correlations are highlighted in bold.

## Data Availability

The data used in this study are freely available on Openneuro (https://doi.org/10.18112/openneuro.ds004840.v1.0.1).

## Abbreviations

The following abbreviations are used in this manuscript:

ECG	Electrocardiogram
EEG	Electroencephalography
EMG	Electromyogram
FDR	False Discovery Rate
FIR	Finite Impulse Response
FSFB	Fundación Santa Fe de Bogotá
HADS	Hospital Anxiety and Depression Scale
ICA	Independent Component Analysis
ICU	Intensive Care Unit
MAR	Music-Assisted Relaxation
MT	Music Therapy
PSD	Power Spectral Density
VAS	Visual Analog Scale
